# A framework for stakeholder identification in concept mapping and health research: a novel process and its application to older adult mobility and the built environment

**DOI:** 10.1186/1471-2458-13-428

**Published:** 2013-05-02

**Authors:** Claire Schiller, Meghan Winters, Heather M Hanson, Maureen C Ashe

**Affiliations:** 1 Centre for Hip Health and Mobility, 6F-2635 Laurel Street, Vancouver, BC V5Z 1M9, Canada; 2 Department of Family Practice, University of British Columbia (UBC), Vancouver, BC V6T 1Z4, Canada; 3 Faculty of Health Sciences, Simon Fraser University (SFU), Burnaby, BC V5A 1S6, Canada

**Keywords:** Stakeholders, Concept mapping, Older adults’ mobility, Built environment, Health

## Abstract

**Background:**

Stakeholders, as originally defined in theory, are groups or individual who can affect or are affected by an issue. Stakeholders are an important source of information in health research, providing critical perspectives and new insights on the complex determinants of health. The intersection of built and social environments with older adult mobility is an area of research that is fundamentally interdisciplinary and would benefit from a better understanding of stakeholder perspectives. Although a rich body of literature surrounds stakeholder theory, a systematic process for identifying health stakeholders in practice does not exist. This paper presents a framework of stakeholders related to older adult mobility and the built environment, and further outlines a process for systematically identifying stakeholders that can be applied in other health contexts, with a particular emphasis on concept mapping research.

**Methods:**

Informed by gaps in the relevant literature we developed a framework for identifying and categorizing health stakeholders. The framework was created through a novel iterative process of stakeholder identification and categorization. The development entailed a literature search to identify stakeholder categories, representation of identified stakeholders in a visual chart, and correspondence with expert informants to obtain practice-based insight.

**Results:**

The three-step, iterative creation process progressed from identifying stakeholder categories, to identifying specific stakeholder groups and soliciting feedback from expert informants. The result was a stakeholder framework comprised of seven categories with detailed sub-groups. The main categories of stakeholders were, (1) the Public, (2) Policy makers and governments, (3) Research community, (4) Practitioners and professionals, (5) Health and social service providers, (6) Civil society organizations, and (7) Private business.

**Conclusions:**

Stakeholders related to older adult mobility and the built environment span many disciplines and realms of practice. Researchers studying this issue may use the detailed stakeholder framework process we present to identify participants for future projects. Health researchers pursuing stakeholder-based projects in other contexts are encouraged to incorporate this process of stakeholder identification and categorization to ensure systematic consideration of relevant perspectives in their work.

## Background

Public health problems are inherently complex, spanning across realms of practice and impacting a variety of stakeholders. The importance of involving stakeholders in health research is increasingly recognized [1-3]. Groups and individuals affected by an issue (such as public health practitioners and community members) possess critical insight that may inform all aspects of the research process, providing valuable input in all stages from setting research priorities, to disseminating and implementing results [4]. The diversity of perspectives that stakeholders possess may be particularly relevant to understanding the complex determinants of health which figure centrally in public health research and practice.

Concept mapping is a mixed-methods technique that facilitates the analysis of stakeholder perspectives. As such, it is a useful tool for understanding complex phenomena in public health [5]. A detailed explanation of the methodology is outlined in Trochim’s seminal work [6] and subsequent publication by Kane and Trochim [7]. In brief, concept mapping integrates group brainstorming and sorting of ideas with quantitative analysis to generate visual representations of concepts. Concept maps reflect the relative importance and relationships between intersecting ideas [7]. A recent review of concept mapping attests to the quality and rigor of the methodology [8]. The review also highlights the increasingly widespread use of concept mapping in health research; of the 69 articles reviewed, over 59% had a public health orientation [8].

In order to implement concept mapping projects, investigators must first identify which stakeholders are relevant to their topic of inquiry. However, this proves to be a challenging task as the literature lacks systematic, practical techniques for identifying stakeholder groups and individuals [9]. In practice, the process is more often guided by intuition and feasibility than structured systematic frameworks [10]. Broad, heterogeneous participation from “relevant people” is generally encouraged in concept mapping projects [7, p.36]. Techniques such as focus groups, semi-structured interviews and snowball sampling (described in more detail below) broadly capture methods of identifying stakeholders, but fail to provide a detailed process required to ensure systematic identification. A challenge, and apparent gap in the literature thus exists with regards to knowing who “relevant people” are in practice.

We encountered the challenge of identifying stakeholders in a concept mapping project on the intersection between older adult mobility with built and social environments [11]. This is an important and emerging area of research; as mobility contributes significantly to the health of older adults, and early evidence suggests that built and social environments interact to impact the ability for older adults to engage in community participation [12]. In this context, we defined: mobility as “the ability of a person to move about and complete physical activities in their community setting” [12]; the built environment as the composite of “urban design, land use and the transportation system”[13]; and the social environment as “social relationships and cultural milieus within which defined groups of people function and interact” [14]. Diverse stakeholder engagement is likely critical to advancing our understanding of this issue, for it has already contributed to other aspects of built environment and physical activity research [15-17]. Yet the literature provides little guidance on how to identify stakeholders in practice and there are no detailed frameworks of stakeholders related to older adult mobility and the built environment. Therefore, in this paper, we present a framework to address this gap and outline a stakeholder identification process that can be applied across public health research, policy and community engagement projects. By discussing the applicability of our framework in the growing practice of concept mapping, we hope to further demonstrate the utility of our work. A brief review of stakeholder theory figures at the forefront of our analysis as it lends clarity to the term “stakeholder” and provides theoretical underpinnings of our framework.

### Stakeholder theory

Freeman is credited with the classic definition of a stakeholder, articulated in his seminal work as “any group or individual who can affect or is affected by the achievements of the organization’s objective” [18, p.46]. This definition reflects the business management context in which the term originated. As a concept, stakeholder extend*s* the responsibilities of business beyond financial investors to other entities that may be affected by a firm’s actions. Most pertinent to other disciplines is the “affect or is affected by” clause which may serve as a criterion to designate individuals or groups as stakeholders. Nuanced variations on the stakeholder definition exist, however Freeman’s is still considered the most broad and balanced [18]. Friedman and Miles identify fifty-five definitions of stakeholder spanning forty years and seventy-five texts; for a more comprehensive comparison of the term, their work should be referenced [18].

In addition to defining the term “stakeholder”, Freeman’s seminal work contributes two other tools for stakeholder identification that may be applied to health research projects. The first is the now common ‘hub-and-spoke’ picture, where stakeholder groups are depicted at the end of spokes emanating from a central firm [19] (See Additional file 1: Figure S1). This figure is an acknowledged oversimplification, as each stakeholder category can be further broken down into more specific groups, however, this visual map is a useful tool for identifying stakeholders [18]. The other contribution is a broader stakeholder analysis process, of which stakeholder identification is only the first step [18]. Subsequent components of stakeholder analysis focus on understanding the interests and stance of various stakeholder groups, and on devising a business management strategy in response. Stakeholder analysis theories offer interesting techniques for prioritizing stakeholders and understanding relationships, but they do not provide practical guidance on how to *identify* stakeholders.

Some additional insight on the practice of stakeholder identification is gleaned from the discussion of stakeholder management issues within *Stakeholders: Theory and Practice*[18]. Notably, the challenge of constructing stakeholder maps is acknowledged, particularly in light of the heterogeneity of interests within stakeholder groups, and the possibility of a single stakeholder belonging to multiple categories [18].

The use of stakeholder analysis has broadened considerably beyond its original application in business management [10]. Environmental resource management, in particular, has embraced this study design, as demonstrated by Reed et al. [20]. The authors build on the theoretical contributions of business management literature, and notably categorize methods employed to identify stakeholders, differentiate between stakeholders, and investigate relationships between stakeholders in practice [20]. Three specific methods of identifying stakeholders are listed, mainly; focus groups, semi-structured interviews, and snowball sampling. These techniques are likely familiar to health researchers, however their application in the explicit context of stakeholder identification is perhaps more novel. In focus groups, a small number of participants brainstorm lists of stakeholders. This method is notably less structured than others, and may be supplemented with interviews of a cross-section of stakeholders [20]. Semi-structured interviews with selected stakeholders are akin to consulting key informants, which is recommended for the analysis of stakeholders by Varvasovsky and Brugha [21]. The snowball sampling technique consists of individuals from initial stakeholder categories identifying new stakeholders and contacts. Possible bias towards the social networks of the first stakeholders should be noted [20], however snowball sampling is nonetheless commonly employed in health management stakeholder analysis [10]. Although these techniques broadly capture methods of identifying some stakeholder group, they do not provide a systematic method for identification in practice.

As discussed in the context of concept mapping above, a challenge and gap in the literature exists in regards to knowing who “relevant people” are. A systematic process for determining which perspectives or stakeholders are relevant is not described in health research methodology. In part this is due to the diversity of contexts and the need to tailor approaches to specific projects. However it also reflects an observation made by Reed et al., [20] that stakeholders are often presumed to be “self-evident” in the literature. In practice it seems intuition and familiarity with a given topic tend to guide identification of stakeholder categories; whether for specific health research projects or broader stakeholder analysis.

A more documented, systematic methodology for stakeholder identification stands to benefit public health research and concept mapping projects by increasing transparency in participant selection and minimizing researcher bias towards familiar groups. Frameworks of stakeholder categories may serve as a starting point for systematic identification of stakeholders, however such frameworks are not commonly cited in the literature. Therefore our aim was to develop a framework of health stakeholder categories and outline its application to older adults’ mobility and built and social environments to identify specific stakeholder groups.

## Methods

To inform the development of our framework we conducted a strategic, focused literature search with particular attention to categories of health stakeholders employed in concept mapping research, so as to inform a separate project conducted by the authors of this paper [11]. The texts *Stakeholders: Theory and Practice*[18] and *Concept Mapping for Planning and Evaluation*[7] served as comprehensive, resources on stakeholder theory and concept mapping methodology. After reviewing relevant citations from these texts, we identified “stakeholder analysis” and “concept mapping” as appropriate search terms. In order to focus our search on health, we limited our search to the health database of Ovid Medline (years 1950 – present). A search in April 2012, identified 68 and 245 citations using our keywords “stakeholder analysis” and “concept mapping” respectively. An additional search of the Cochrane Database for “stakeholders” returned no completed reviews. We then reviewed retrieved articles for relevance to older adult mobility and the built environment in search of applicable stakeholder frameworks.

Identified categories of health stakeholders informed the organization of our framework, however they did not provide sufficient guidance on how to adapt the classification to specific public health contexts, such as the intersection of older adults’ mobility with the built and social environments. To address this gap in the literature and facilitate stakeholder identification, we present a detailed description of the steps employed in this project in addition to the final framework.

Broadly speaking, our stakeholder framework was created through an iterative process of revising stakeholder categories to encompass individual stakeholders deemed important by literature and experienced informants. The framework is presented as a visual representation and classification of groups and individuals related to the intersection of older adult mobility with the built and social environments.

Varvasovszky and Brugha recommend a mixed team of internal and external analysts to conduct stakehodler analysis [21]. Our initial chart was thus created by one author (CS) who had little *a priori* knowledge of the relation between older adult mobility and the built and social environment, to increase objectivity and benefit from an external, theory driven identification of stakeholders. The scope and methods of analysis were derived in consultation with all authors (experienced in this area), and the final stakeholder framework reflects collective expertise.

To enhance the project with practice-based insight, four expert informants reviewed and provided feedback on an initial draft of the stakeholder framework. Expert informants were professionals with knowledge of the field and represented policy makers, researchers, practitioners and service providers, and were chosen based on the individuals’ expertise and prior collaboration. All worked across disciplines but had primary training or worked professionally in the fields of health or social services. Expert informants were asked to review the stakeholder framework and provide open-ended feedback on the organization of stakeholder groups and identification of missing stakeholders. We collected comments via email in accordance with a consent protocol approved by the Simon Fraser University Department of Research Ethics (File #:2012s0331). The final stakeholder framework incorporated recommendations from the expert informants.

## Results

### Creation process

**Figure 1:**
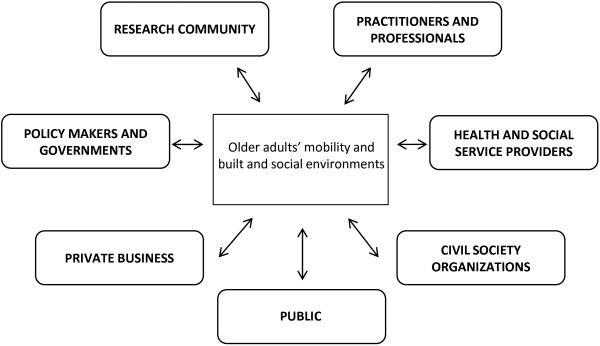
Framework of stakeholder categories related to the intersection of older adults’ mobility with built and social environments.

**Figure 2:**
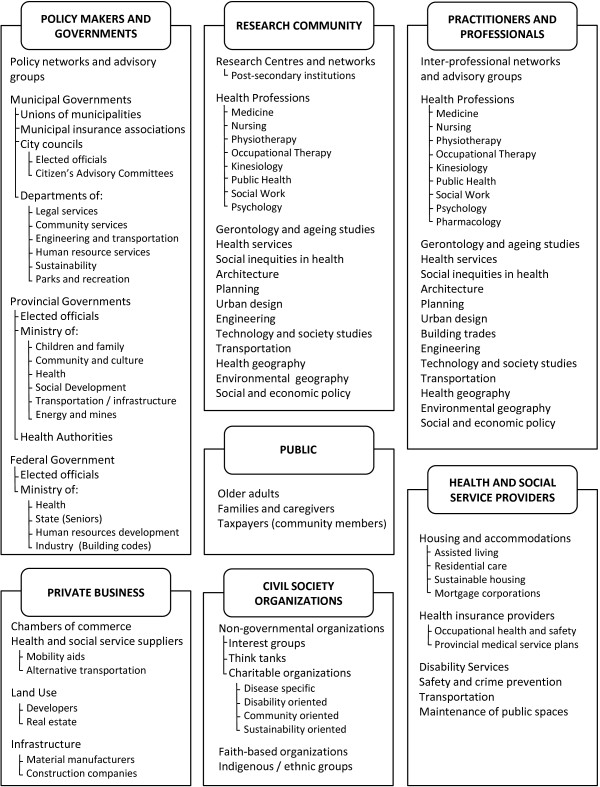
Detailed chart of stakeholders, expanding framework of stakeholder categories related to the intersection of older adult mobility with built and social environments.

An account of the systematic process employed in this project precedes the final framework (Figures 1 and 2), providing justification for the stakeholders identified and, of particular value, guidance for others undertaking a similar task. The iterative process was articulated as the following series of three main steps:

1. Identify a relevant framework of stakeholder categories

Based on an iterative search of the literature, no frameworks of stakeholder categories specific to older adult mobility, the built environment, or social environments were identified. Three classifications of health stakeholders were found [22-24]. The most concise and explicit articulations of health stakeholders for concept mapping was listed by Trochim and Kane [23]. Although not presented as a formal framework for stakeholder categorization, Trochim and Kane identified relevant health stakeholders including the public, health professionals, health administrators, policy makers and politicians, and the research community. A second concept mapping project on chronic disease prevention in Canada used the broad categories of researchers, practitioners, and policy specialists to classify health stakeholders [22]. In a third example of stakeholder analysis in health research, a comprehensive list of stakeholders is presented by Future Health Systems: Innovations for Equity [24]. Within the context of health systems research in developing countries, the authors recommend systematic consideration of the following eleven stakeholder categories; beneficiaries, central government agencies, ministry of health, local governments, financiers, civil society organizations, health governing boards, provider organizations, professional organizations and health workers, unions, and suppliers [24].

The categories of health stakeholders identified by Trochim and Kane [23] were adapted in this project as they encompassed most other categories while maintaining an element of simplicity. These categories included the public, health professionals, health administrators, policy makers and politicians, and the research community. As our stakeholder framework evolved, new categories of stakeholders were added and some were renamed. For example, ‘Health providers’ (identified by Hyder et al., [24]) replaced health administrators as a main category of stakeholders and ‘Health professionals’ was broadened to ‘Professionals and practitioners’.

2. Identify specific stakeholder groups:

(i) Begin with relevant research disciplines

We first discerned relevant research disciplines to initiate the identification of specific groups of stakeholders within each category. This step was greatly informed by an evidence review published by co-authors [12]. For the purpose of stakeholder identification, a list of relevant research disciplines was generated based on the academic affiliations of authors of the papers in the review and the types of journals in which they were published. Twenty-one disciplines were identified in this manner, and were added to the framework as stakeholder groups within the categories of ‘Research community’, and ‘Professionals and practitioners’.

Subsequently, stakeholder groups related to these disciplines within other categories were added to the framework. For example, community planners were identified as important members of the research community based on the critical literature review. Corresponding professional planners were added to the ‘Professionals and practitioners’ category, and municipal government branches responsible for community planning were added to the category of ‘Policy makers and governments’.

(i) Supplement with collaborative networks

Internet searches for working groups and collaborative networks related to older adults’ mobility and the built and the social environment further facilitated the identification of specific stakeholder groups. A list of ‘potential partners’ identified by the Canadian Coalition Linking Action and Science for Prevention was particularly useful resources to guide our search [25]. In many instances the networks could be considered relevant stakeholders in and of themselves, and were added to the framework. However the collective interest of such groups may differ from the individual member organizations, thus these smaller stakeholder groups were also individually added. At times, stakeholders identified through this process did not readily fit within the broader categories, leading to revisions of the stakeholder categories and reorganization of the framework. A notable example was the addition of a category for ‘Private business’, not originally included in the categories adapted from Trochim and Kane [23].

3. Solicit feedback from expert informants

Feedback from four expert informants was collected to ensure that the stakeholder framework reflected the realities of practice and included important stakeholder groups that may have been missed in our search of the literature. Expert informants collectively represented policy makers, researchers, practitioners and service providers. All invited informants participated and suggested improvements on a draft of the stakeholder framework.

Overall, informants expressed agreement with the stakeholder categories and organizational structure of the framework. Each informant identified some specific stakeholder groups and organizations to be added, and re-categorization of a few specific organizations was suggested. The feedback was particularly helpful in further developing the categories of ‘Civil society organizations’ and ‘Private business’, as these were the stakeholder categories most poorly informed by the literature. Classifying non-governmental organizations according to the services provided, helped structure the ‘Civil society organization’ category. It also helped identify ‘Private business’ stakeholders and ‘Health and social service provider organizations’ that work to support similar causes. For example numerous civil society organizations provide support for people with disabilities; however government agencies (classified as ‘Health and social service providers’) also address these needs, as do private businesses that provide supplies and disability-oriented services. These additional stakeholders were incorporated into the final framework, and informed development of sub-categories. The practice-based insight of expert informants also helped identify some specific government departments, collaborative networks, and additional grey literature on related older adult programs [26].

### Final stakeholder framework

Feedback from the expert informants and co-authors guided revisions to the stakeholder framework resulted in the final version shown in Figures 1 and 2. The condensed version of the framework shown in Figure 1 highlights the general categories of stakeholders related to the intersection of older adult mobility with the built and social environments. These include: (1) Public, (2) Policy makers and governments, (3) Research community, (4) Practitioners and professionals, (5) Health and social service providers, (6) Civil society organizations, and (7) Private business. This figure is further grounded in stakeholder theory as it reflects Freeman’s original ‘hub and spoke’ diagram [19].

Figure 2 captures the rich contributions of this process, as it elaborates on these categories, identifying subset groups of relevant stakeholders. Although specific organizations are not named in this publication, the iterative process of identifying specific organizations and determining which broader categories of stakeholders they belonged to was critical to the creation process of the framework. Development of new categories spurred the identification of specific groups, just as the identification of specific groups informed the development of new categories. The large number of stakeholders identified in Figure 2 demonstrates the diversity of individuals and organizations related to the intersection of older adult mobility with built and social environments.

## Discussion

We present a framework of stakeholder categories and applied it to the intersection of older adult mobility with the built and social environments. The result was a comprehensive, framework of stakeholder categories that can be used to understand older adult mobility. Furthermore, the novel process of stakeholder identification can be applied across health disciplines in other concept mapping projects to understand various matters of public health concern. For example, one area of research to which our framework may be readily adapted is the growing study of environmental and policy approaches for promoting physical activity [16,17].

The details of the process of stakeholder identification are of particular value to the literature. The aim of systematic identification of stakeholders is to ensure comprehensive representation of diverse perspectives on an issue. Poorly structured or unsystematic stakeholder identification risks missing valuable perspectives or limiting participation to groups readily known to health researchers. Often marginalized groups and the public’s perspective is lacking from academic literature [20]. Without a framework or structured method of identification, omissions may go undetected. Our framework does not eliminate the risk of omissions, but is a guide to identifying stakeholder groups and helps identify which perspectives may be missing. Our review of stakeholder theory and concept mapping literature suggested three general techniques for stakeholder identification: brainstorming, key informant interviews, and snowball sampling. These techniques broadly capture methods of identifying stakeholders, but they fail to provide a detailed process required to ensure systematic identification of relevant stakeholders. Another approach is to rely on existing frameworks of stakeholder categories to provide a starting point for systematic identification; however such frameworks – particularly as they relate to health – were not commonly cited in literature.

In applying the results of this study to future stakeholder-based projects, we encourage public health researchers and practitioners to use a framework of stakeholder categories to inform their selection of participants. At a minimum, categories of stakeholders add a level of structure to subsequent brainstorming and facilitate the identification of missing groups. The seven categories of stakeholders developed in this study (Public, Policy makers and governments, Research community, Professionals and practitioners, Health and social services providers, Civil society organizations, and Private business) may serve as a template for health-related projects and may be adapted to specific areas of research. Even if all the groups identified are not invited to participate, these missing perspectives may be acknowledged as a limitation of the final results, or justification for their exclusion clearly stated. The process of systematic stakeholder identification can thus increases the methodological rigour of concept mapping and other stakeholder-based projects.

In applying this framework to future research on older adult mobility and the built and social environment, stakeholders identified in Figure 2 can be further specified to reflect the regional context of interest. For example, specific provincial, state, or municipal stakeholders could be identified depending on the scope of study. Initially a national scope was proposed for the concept mapping project that motivated this project. However, as the stakeholder categories of our framework developed, a provincial focus was adopted to provide better context for the stakeholder chart and a more feasible scope for the project.

One of the biggest challenges in developing a framework of stakeholders is representing a complex, intersectional issue in a simplistic model. Distinctions between researchers, professionals, and policy makers, for example, are intuitively convenient but blurred in practice. Many disciplines, and even individual people, fulfill a multiplicity of roles and could be classified under several stakeholder groups. The task of identifying and organizing stakeholder groups within categories thus proved to be a challenging conceptual exercise, and more than mere ‘filling in the blanks’ of a generic framework. It is our intent that this framework and process of stakeholder identification will enable other health researchers to complete the task more effectively.

Which stakeholders should and do participate in any stakeholder-based project depend on a number of factors. Thoughtful identification of stakeholders does not in and of itself guarantee comprehensive participation in public health and concept mapping projects; recruitment and engagement strategies will also be required to ensure participation of desired groups. Prioritization of stakeholders is also often required, and this may limit the breadth of participation. We, like others[20], caution researchers against prematurely limiting the scope of identified stakeholders, as even remotely affected groups may prove to be important contributors. Concern of identifying too many or irrelevant stakeholders should not inhibit an initial thorough assessment of stakeholder groups. When subsequent boundaries must be drawn, it should be on well-founded, clearly articulated criteria [20].

This project had an explicit health focus, as older adult mobility was the main outcome of interest. Prioritizing health helped define the scope and refine the analytic approach used to create the framework. Recognizing, as others have [21], that researchers are often stakeholders in the issues under study, we took steps to enhance objectivity in developing the framework before starting other projects. For example, in order to facilitate a systematic, literature driven process of stakeholder identification, the initial framework was created by a single author (CS) previously external to the project. This process was complemented by feedback from co-authors and external expert informants with diverse expertise to minimize the bias of any one perspective. Although this framework was developed within the regional context of British Columbia, we have provided direction on how it can be generalizable to other settings. We can also attest to the utility of the framework in practice. We relied on it to identify and invite stakeholders from each of the seven categories to participate in our concept mapping project on older adult mobility and the built environment [11].

As with any review of the literature, our work is limited by its inability to report on newly published articles. Since April 2012 when we conducted our literature search, 13 new citations for “stakeholder analysis” and 56 on “concept mapping” were indexed in Ovid Medline. This increase in concept mapping publications, however, reflects a growing interest in this type of research and provides all the more justification for why a framework of identifying stakeholders is timely and of value.

## Conclusion

This paper provides guidance for those undertaking stakeholder-based projects on ways to increase the methodological rigour of participant selection. The stakeholder framework presented is of direct relevance to the study of older adult mobility and the built and social environments, but is also of broader value to anyone seeking stakeholder involvement and in particular for concept mapping projects. This process of stakeholder identification may be adapted and applied in other public health contexts to gain a broader understanding of complex issues. For those to whom the intersection of older adults’ mobility with built environments is an interest, the detailed framework and seven categories of stakeholders may help identify important collaborators to engage in future research.

As health research agendas are increasingly shaped by stakeholder involvement, critical reflection on who constitutes a stakeholder is warranted. Others undertaking stakeholder-based initiatives are encouraged to systematically identify participants based on explicit categorization frameworks. This added rigour in the initial stages of stakeholder identification stands to enhance our understanding of complex public health issues, and ensure that critical perspectives are not overlooked.

## Competing interests

The authors declare that they have no competing interests.

## Authors’ contributions

CS, MA and HH contributed to the conception of the project, and all authors contributed to the study design. CS completed data collection and analysis for initial framework in partial fulfillment of a Master’s Degree. CS, MA, HH, and MW contributed to refined versions of the framework. All authors contributed to manuscript drafts and reviewed the final manuscript.

## Pre-publication history

The pre-publication history for this paper can be accessed here:

http://www.biomedcentral.com/1471-2458/13/428/prepub

## Supplementary Material

Additional file 1: Figure S1 Stakeholder Map of a Very Large Organization [19], p.55. Reprinted with permission from Cambridge University Press. Click here for file
