# Supplementary figures and images for: A framework for stakeholder identification in concept mapping and health research: a novel process and its application to older adult mobility and the built environment
Source: BMC Public Health. 2013 May 2;13:428. doi: 10.1186/1471-2458-13-428 (PMC3653754; doi:10.1186/1471-2458-13-428)

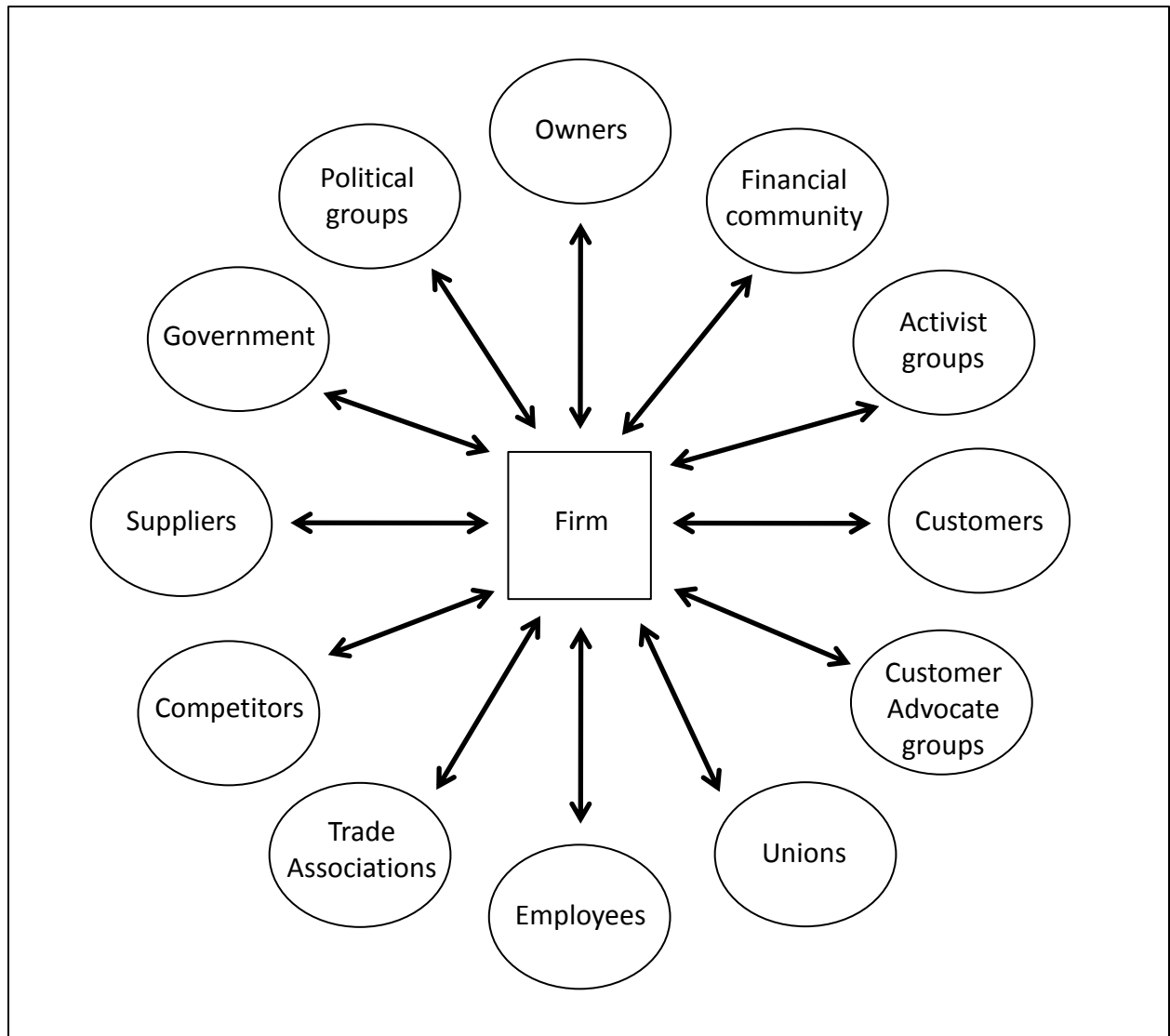

Supplement: Additional file 1: Figure S1 — Stakeholder Map of a Very Large Organization [19], p.55. Reprinted with permission from Cambridge University Press. [file 1471-2458-13-428-S1.pdf]
